# Growth, Cell Division, and Gene Expression of *Escherichia coli* at Elevated Concentrations of Magnesium Sulfate: Implications for Habitability of Europa and Mars

**DOI:** 10.3390/microorganisms8050637

**Published:** 2020-04-27

**Authors:** Sudip Nepal, Pradeep Kumar

**Affiliations:** 1 Department of Physics, University of Arkansas, Fayetteville, AR 72701, USA; snepal@uark.edu; 2 Microelectronics and Photonics Graduate Program, University of Arkansas, Fayetteville, AR 72701, USA

**Keywords:** habitability of Europa and Mars, response of bacteria to high salinity, hyperosmolar stress, magnesium sulfate, *Escherichia coli*

## Abstract

We perform quantitative studies of the growth, death, and gene expression of *Escherichia coli* in a wide range of magnesium sulfate (MgSO${}_{4}$) concentrations (0–2.5 M). Elevated concentration of MgSO${}_{4}$ causes the inhibition of cell growth, leading to an increase in the population doubling time. We find that cells exhibit three distinct morphological phenotypes—(i) normal, (ii) filamentous, and (iii) small cells at 1.25 M MgSO${}_{4}$. Filamentous cells arise due to the lack of cell division, while the small cells arise due to the partial plasmolysis of the cells. We further find that cell death starts for salt concentrations >1.25 M and increases with an increasing concentration of MgSO${}_{4}$. For salt concentrations ≥1.66 M, the growth of cells stops and all the cells become smaller than the control cells, suggesting the plasmolysis of the population. Cells grown at salt concentration up to 2.07 M are reversible in both the growth rate and morphology upon the removal of the salt stress. The time scale of reversibility increases with increasing salt concentration. Finally, we investigate the expression of an osmotically inducible gene (*osmC*), genes involved in magnesium transport (*corA*), sulfate transport (*cysP*), and osmotically driven transport of water (*aqpZ*). We find that a high concentration of magnesium sulfate leads to the upregulation of *cysP* and *osmC*.

## 1. Introduction

Europa, one of the Galilean moons of Jupiter, is a significant candidate in the search for life beyond Earth, due to the presence of a global subsurface liquid water ocean in contact with a dense rocky core [1,2,3,4,5]. The mean surface temperature of Europa is about 100 K, hence, the surface is a thick icy crust with the global ocean running 100–200 km deep underneath it, with hydrostatic pressure reaching 130–260 MPa at the seafloor [6]. A recent study using spatially resolved spectra of Europa’s surface argues that MgSO${}_{4}$ is a radiation product, rather than a constituent of Europa’s brine, and suggests an abundance of chlorides of sodium and potassium in Europa’s ocean [7]. However, other studies, using a combination of geochemical modeling and Galileo’s Near Infrared Mapping Spectrometer observation of the surface, suggest that Mg${}^{2+}$, SO${}_{4}^{2-}$, Na${}^{+}$, and Cl${}^{-}$ may be the most abundant ions in the ocean [8,9]. In order to constrain the ionic composition based on surface measurements, experiments on various aqueous compositions of salts were performed at low temperatures [10]. This study suggests that if the endogenic origin of sodium sulfate and magnesium sulfate is confirmed, then it would imply an ocean with a low pH and rich in magnesium and sulfate and poor in sodium [10]. These geochemical models further predict that the concentrations of Mg${}^{2+}$ and SO${}_{4}^{2-}$ can be as high as 2.9 M and 3.6 M, respectively, depending on the temperature [8,9]. The presence of hygroscopic salts of Mg, Ca, Fe, and Na in Mars regoliths is well established [11,12,13,14]. These hygroscopic salts could retain water, forming liquid water brines [15]. According to some studies, the sulfate concentration in the regolith could be as high as 30% by weight [14,16]. This would entail that, for any organism to thrive on Europa or Mars, it must be adapted to high concentrations of magnesium sulfate along with other environmental factors. These conditions are not unknown to the terrestrial organisms. Many organisms on Earth thrive in harsh conditions such as high pressure, extreme temperatures, pH, salinity, and a combination of them [17,18,19,20]. Though rare, epsomic environments exist on Earth, such as the Basque Lakes and the Spotted Lake in Canada and the Qaidam Basin in China, that are rich in MgSO${}_{4}$ [21,22,23,24]. Metagenomics studies of the microbial community of the Spotted Lake suggests an abundance of Proteobacteria, Firmicutes, and Bacteroidetes, as well as diverse extremophiles [25]. Another metagenomics study has investigated the change in the microbial community in soil samples from the Qaidam Basin as a function of Mg${}^{2+}$ concentration in the soil [26]. They found an abundance of Firmicutes and Proteobacteria at a high concentration of Mg${}^{2+}$. Both Firmicutes and Proteobacteria subgroups have a large number of moderate and extreme halophiles. Recent studies have demonstrated the growth of salinotolerant bacteria isolated from the Great Salt Plains (OK), Hot Lake (Oroville, WA, USA), and Basque Lakes under high concentrations of magnesium sulfate [27,28,29,30]. While these studies reveal the microbial community structures in hypersaline environments and their potential growth in those environments, they do not provide information regarding the adaptation mechanisms of these microbes to these environments. The investigation of cellular responses of a non-salinotolerant microbe in hypersaline environments provides a window into bottleneck cellular functions more prone to fail when exposed to these conditions. Studies on the effect of high hydrostatic pressure on a halotolerant bacterium, *Escherichia coli* (*E. coli*), suggest that this bacterium can withstand very high pressures in a temperature dependent way [31,32]. An interesting question arises whether these cells can also tolerate high concentration of magnesium sulfate, presumably the most abundant salt on Europa and Mars.

Large concentration of ions create dual stress, both ionic and osmotic. While ions are needed for cellular functions, the presence of large amounts of ions may affect the structure, stability, and functionality of proteins [33]. At low concentrations of salt, proteins are soluble in water, while a high concentration of salt leads to the precipitation of proteins, a phenomenon known as salting out. The binding affinity of charged ligands to DNA is sensitive to salt concentration [34]. Liquid water and its activity is essential for terrestrial life. The addition of salt decreases the water activity [35]. The investigation of the limiting water activity for microbial life suggests that both prokaryotic and eukaryotic life forms cannot grow at water activities lower than ≈0.6 [36]. A halotolerant bacterium, *E. coli*, experiences several challenges under high salt concentration. Studies of the effects of the most common salt, sodium chloride, on *E. coli* suggest that the viability of cells does not change up to 0.51 M. The viability of the cells decreases upon the further increase of the salt concentration [37]. Studies of osmotic shock exerted on the bacterial cells indicate the active regulation of cell volume in response to the high concentration of salt [38]. Hyperosmolarity of media results in the plasmolysis of cells [39,40]. Cells regulate expression of many genes in response to the changes in their surroundings. Earlier studies have identified a number of genes involved in osmoregulation and osmoadaptation of cells. Sigma factor RpoS is a global transcriptional regulator of various genes in response to different stresses including heat, oxidative, and osmotic stress [41]. For example, *osmC*, an osmotically inducible gene, is upregulated under high osmolarity [42]. The transport of water from the outside to the inside of a cell, as well as from the inside of a cell to the outside, is extremely important for osmolarity regulation. Pure diffusion of water is too slow for cells to rapidly respond to external osmolarity changes, and hence cells require the facilitated transport of water. AqpZ, an aquaporin water channel, is shown to mediate both an inward and outward flux of water in response to changes in external osmolarity [43,44]. Under hyperosmotic conditions, *aqpZ* is downregulated [45]. The primary Mg${}^{2+}$ transporter in *E. coli*, CorA, is expressed constitutively and is essential for maintaining Mg${}^{2+}$ homeostasis inside the cells [46,47,48]. In the presence of low cytoplasmic levels of Mg${}^{2+}$, it is in the open state configuration, while in the presence of high cytoplasmic levels of Mg${}^{2+}$, it is in the closed state configuration. Sulfur is essential for making methionine and cystine amino acids and must be taken from the environment in inorganic or organic forms. Thiosulfate binding protein and a part of the ABC transporter complex, CysP, is involved in the transmembrane transport of sulfate and thiosulfate [49,50].

A large body of literature exists on the effect of both low [35,51,52] and high salt concentration [40,53,54,55] on cellular functions of bacteria. Most of these studies have taken sodium chloride or potassium chloride as models of salt. The effect of MgSO${}_{4}$ on bacterial cells is poorly understood. In order to explore the cellular response to a high concentration of magnesium sulfate, we study the cell growth and death, morphology, and gene expression of a number of genes involved in osmolarity regulation and the transport of magnesium and sulfate of a halotolerant bacterium, *E. coli*, at different concentrations of MgSO${}_{4}$. The paper is organized as follows. In the section Materials and Methods, we describe the experimental protocols and data analysis approaches. In the section Results, we describe the results followed by conclusion in the Discussion section.

## 2. Materials and Methods

### 2.1. Cell Culture and Media

Wild-type *Escherichia coli* K-12 strain MG1655 was obtained from the Coli Genetic Stock Center located at the Yale University, USA. Cells were cultured in M9 media with the supplement of 0.4% glucose and 0.4% succinate as carbon sources containing various concentrations of anhydrous MgSO${}_{4}$. The pH of the media decreases with increasing concentration of MgSO${}_{4}$ and is 5.3 at 1.25 M of salt. The media was filter-sterilized by passing it through a 0.22μ m filter (Thermo Fisher Scientific, Carlsbad, CA, USA). The minimum required concentration for the growth of cells in M9 medium is 2 mM, and we will refer to it as the control media. Solid media, M9-agar, was prepared by adding 1.5% agar (BD Difco, Franklin Lakes, NJ, USA) in the liquid media. Bacterial cells were first cultured in a petri dish containing M9-agar media and incubated at 37 °C for 16 h. A single colony from the petri dish was picked and subsequently grown in liquid M9 media at 37 °C in a shaking incubator until the optical density (OD${}_{600}$) reached 0.5±0.1. Subsequently, the bacterial sample was diluted to OD${}_{600}$≈0.10 in liquid M9 media containing different concentrations of MgSO${}_{4}$. The optical density of cells was measured every 30 min using Lambda Bio UV/VIS spectrophotometer (PerkinElmer, Waltham, MA, USA). For the salt concentrations at which cells do not exhibit any growth, the cells were cultured for 15 h, and were used for analyses.

### 2.2. Imaging and the Image Analysis

Phase-contrast images of cells were acquired using a SPOT imaging camera mounted on a Nikon EFD-3 microscope with a 40X objective, immediately following the experiments to minimize the error in the morphology of the bacterial cells. Images were taken without any bias. Images thus obtained were converted into binary images using ImageJ [56]. Binary images were subsequently analyzed using a custom MATLAB code to extract cell length.

### 2.3. Transmission Electron Microscopy (TEM)

Cells grown at different concentrations of MgSO${}_{4}$ were centrifuged at 2000 rpm for 8 min. The supernatant was discarded, and the pellets were resuspended in phosphate buffer saline (PBS). A TEM grid was then dipped into the sample for two minutes. Then the grid was submerged in 2% lead acetyl for two minutes. The sample was then dried for 24 h in a petri dish at room temperature and was visualized under TEM.

### 2.4. Cell Death Assay

Bacterial samples were washed twice in PBS and were then diluted in PBS with final OD${}_{600}$ ≈ 0.1. 20 ng/mL of propidium iodide (PI) was added to the solution and incubated in the dark at the room temperature for 20 min. Samples were then washed twice with PBS and were used to obtain the fluorescence intensities of cells using BD Ariafacs Fusion cell cytometer (BD Bioscience, Franklin Lakes, NJ, USA).

### 2.5. Reversibility in a Liquid Media

In order to investigate the time that it takes for cells to start behaving like normal cells upon removal of the salt stress, we performed reversibility studies of growth and morphology. Specifically, samples of cells starting with OD${}_{600}\approx 0.10$ were grown at different concentrations of magnesium sulfate in M9 media till the OD${}_{600}$ reaches 1.0. Subsequently, cells were diluted to OD${}_{600}\approx 0.04$, and were inoculated in fresh M9 media with 2 mM magnesium sulfate. The optical density of the samples was measured every 30 min until the OD${}_{600}$ reaches 1.2 (five generations). Samples were again diluted to OD${}_{600}\approx 0.04$, and the growth measurements were performed. We call each of these cycles of dilution and growth, a passage. At the end of each passage, images of the cells were obtained and analyzed as described in the Image Analysis section. These passages were repeated for all samples until both the growth rate and the morphology of cells became comparable to control cells. Lag time, the time it takes before the cells start growing exponentially upon removal of the salt stress, was also recorded for samples grown at different concentrations of magnesium sulfate.

### 2.6. Primers and RT-qPCR

The effect of magnesium sulfate on the comparative expression of different genes of *E. coli* was studied using a real-time quantitative PCR (RT-qPCR), as described in a previous study [57]. The primers used in this study are listed in Table 1. Primers were designed on the basis of published GeneBank data using Primer 3 software (National Center for Biotechnology Information) and were purchased from Integrated DNA Technologies (Coralville, IA, USA). The primers were tested using NCBI-Primer BLAST, melt curve analysis, and in silico PCR [58]. The amplicon length of each primer is 150 bp. For the bacterial sample preparation, a single colony was picked from solid media and inoculated in liquid M9 media. The sample was grown until OD${}_{600}$ reached 0.8 and was resuspended in a new media by 16 fold dilution. A respective amount of salt was added to the media for the sample grown at 1.25 M salt concentration. Cells were grown till the mid-exponential phase (OD${}_{600}\approx 0.6$) and total RNA was extracted using RNeasy mini kit (Qiagen, Germantown, MD, USA). The total RNA sample was treated with DNAse (Thermo Fisher Scientific, Carlsbad, CA, USA) and converted to complementary DNA (cDNA) using iScript cDNA synthesis kit (Bio-Rad, Hercules, CA, USA). The cDNA template was amplified using iQ SYBR Green supermix (Bio-Rad, Hercules, CA, USA) and the respective primers. The amplification was performed on Quant Studio 3 real-time PCR system (Applied Biosystems, Thermo Fisher Scientific, Carlsbad, CA, USA). The gene expression data were normalized to 16S *rRNA* and the relative expression, $2^{-\Delta \Delta \mathrm{Ct}}$, was calculated over three biological and two technical replicates. The error bar on the relative expression was estimated from the standard error on ΔCt for each gene.

## 3. Results

### 3.1. Cell Growth and Cell Death at Elevated MgSO${}_{4}$ Concentration

**Figure 1:**
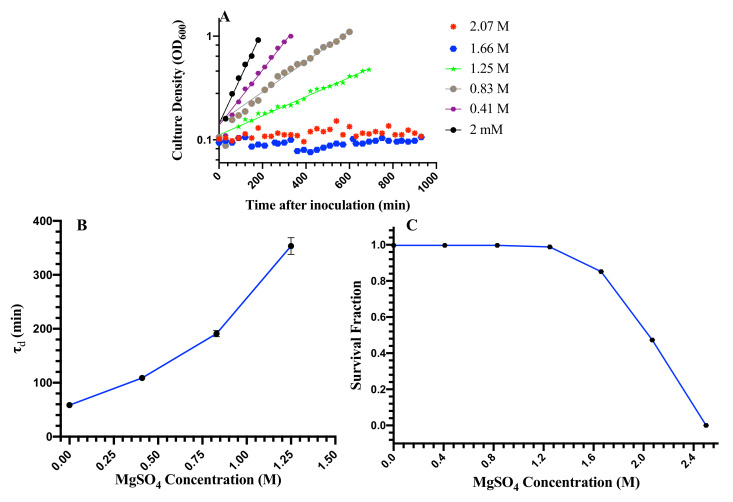
Growth and death of *E. coli* at different MgSO${}_{4}$ concentrations. (**A**) Growth curves of bacteria at different concentrations of magnesium sulfate. (**B**) The population double time, $\tau_{d}$, as a function of magnesium sulfate concentration. Population doubling time increases with increasing concentration of magnesium sulfate. Since cells do not exhibit any growth for salt concentrations greater than 1.25 M but undergo cell death, we do not measure the population doubling time for these concentrations. (**C**) The survival fraction of cells as a function of magnesium sulfate concentration. Survival fraction does not change for concentrations lower than 1.25 M. Beyond this salt concentration, the survival fraction drops sharply.

In order to investigate the effects of different concentrations of MgSO${}_{4}$ on cell growth and cell death, cells were grown in M9 media with the supplement of six different concentrations of MgSO${}_{4}$, 2 mM, 0.41 M, 0.83 M, 1.25 M, 1.66 M, 2.07 M, and 2.50 M. All the experiments were performed at temperature, T = 37 °C. The optical density (OD${}_{600}$) of cultured samples was measured every 30 min. The OD${}_{600}$ thus obtained was used to analyze the growth of the cells. Figure 1A shows the growth curves of the cells for all the concentrations of magnesium sulfate studied here. To characterize the growth rate, we measured the population doubling time, $\tau_{d}$, by fitting an exponential of the form ${\mathrm{OD}}_{600}\left(t\right)={\mathrm{OD}}_{600}\left(0\right)2^{\frac{t}{\tau_{d}}}$ through the growth curves. The population doubling time, $\tau_{d}$, as a function of salt concentration is shown in Figure 1B. The population doubling time increases with an increasing concentration of magnesium sulfate. Since cells do not exhibit any growth for salt concentrations greater than 1.25 M but undergo cell death, we do not measure the population doubling time for these concentrations.

We next studied the effect of magnesium sulfate on the viability of cells. Cells were grown in liquid cultures with starting OD${}_{600}=0.10$, and a small volume of the cells at OD${}_{600}=1.0$ was taken out and stained with PI. Fluorescence intensities of about 120,000 cells were acquired using a fluorescence cell cytometer. The fluorescence intensity data were analyzed to get the survival fraction of the population for each sample. Figure 1C shows the survival fraction of cells as function of salt concentration. Cell death is negligible and comparable to control cells for salt concentrations ≤1.25 M. Beyond 1.25 M, the rate of cell death increases monotonically. We do not observe the growth of cells for salt concentrations ≥1.66 M and find a population collapse of the cells after 30 h in 2.5 M magnesium sulfate. These results suggest that *E. coli*, a halotolerant bacterium, can withstand up to 1.25 M magnesium sulfate, without death.

### 3.2. Cell Division, Cell Length, and Cellular Heterogeneity

**Figure 2:**
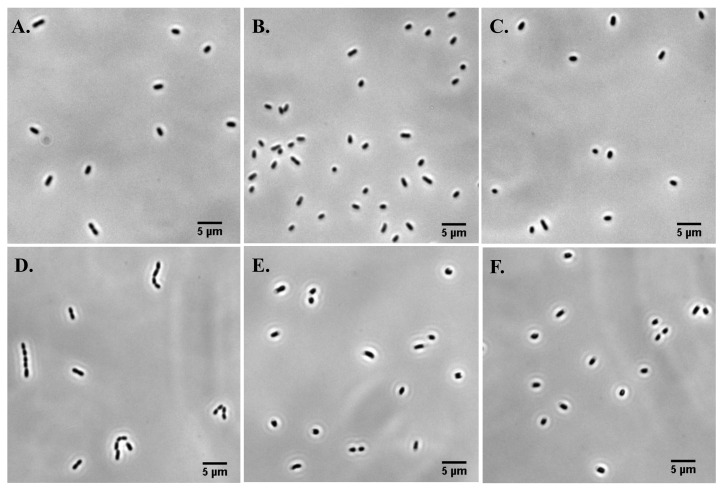
Representative images of *E. coli* at MgSO${}_{4}$ concentrations of (**A**) 2 mM, (**B**) 0.41 M, (**C**) 0.83 M, and (**D**) 1.25 M, respectively. The images were taken when the sample OD${}_{600}=1$. (**E**,**F**) are the images of the cells grown for 15 h in MgSO${}_{4}$ concentrations of 1.66 M and 2.07 M, respectively.

We next studied the effect of different concentrations of magnesium sulfate on cell morphology. Cells were cultured in liquid media with different concentrations of magnesium sulfate until the optical density reached 1. Cells were then taken out and a small volume (0.5μ L) was placed on multiple slides and imaged immediately under the microscope. Figure 2 shows the representative images of bacterial cells grown at MgSO${}_{4}$ concentrations of 2 mM (control), 0.41 M, 0.83 M, 1.25 M, 1.66 M, and 2.07 M. The cell length decreases with the increasing concentration of magnesium sulfate except at 1.25 M. At 1.25 M concentration, population of the cells exhibits three different phenotypes—(i) cells comparable to the length of control cells, (ii) cells smaller than the control cells, and (iii) filamentous cells. In the case of the filamentous cells, cell septum site is visible but cell division has not occurred. Since these filamentous cells have not divided, we assume each of these cells to be a single cell (see Figure 2D).

**Figure 3:**
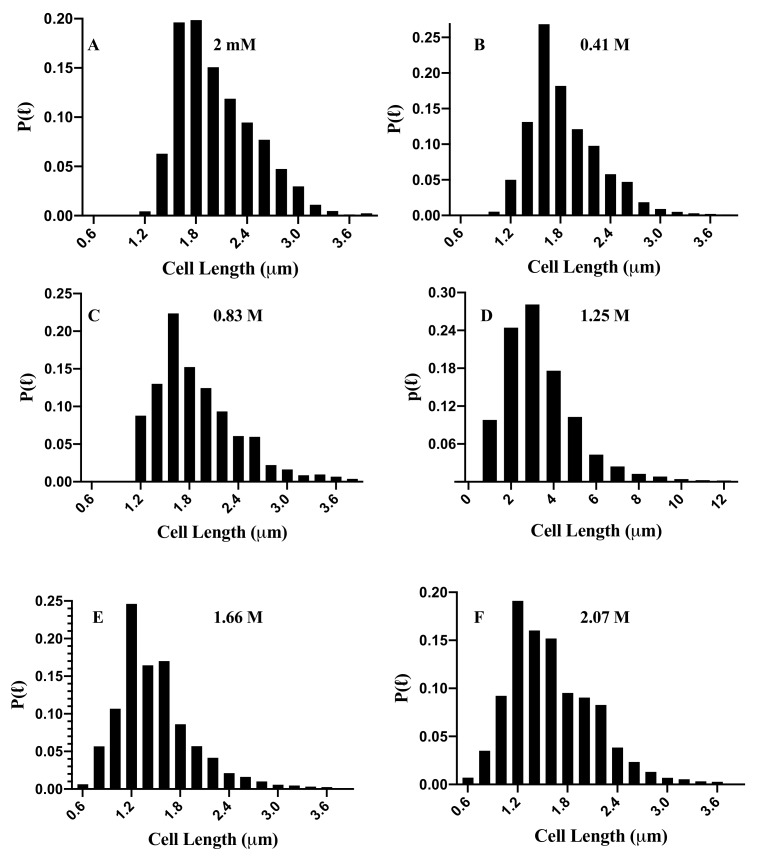
Probability distribution, P(ℓ), of cell length of *E. coli* at MgSO${}_{4}$ concentrations of (**A**) 2 mM, (**B**) 0.41 M, (**C**) 0.83 M, (**D**) 1.25 M, (**E**) 1.66 M, and (**F**) 2.07 M, respectively. The *x*-axis range is the same for all the panels except panel D. For salt concentrations ≤0.83 M, the probability distributions are similar but shift to lower values of length. Cell length exhibits large heterogeneities at 1.25 M of salt concentration, leading to a long-tailed distribution. Note that filamentous cells are counted as single cells. For concentrations >1.25 M, there is cell death, and the probability distribution is over both dead and live cells.

**Figure 4:**
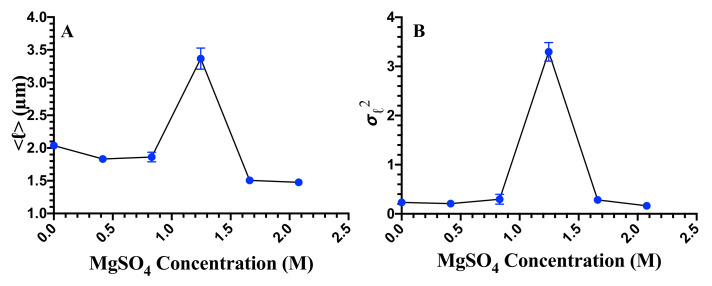
(**A**) Average length, $\langle \ell \rangle$, and (**B**) variance, $\sigma_{\ell }^{2}$, as a function of MgSO${}_{4}$ concentration. Since for concentrations >1.25 M, cells undergo death, and therefore, both the average and the variance of cell length exhibit a maximum at 1.25 M.

Image data obtained above were then analyzed to extract the cell length. In Figure 3, we show the probability distributions, P(ℓ), of cell length, *ℓ*, for 2 mM (control), 0.41 M, 0.83 M, 1.25 M, 1.66 M, and 2.07 M concentrations of magnesium sulfate. For salt concentrations ≤0.83 M, the distributions are similar to control cells but are slightly shifted to smaller values of the length. For salt concentration 1.25 M, the distribution is long-tailed due to the presence of filamentous cells in the population. The mean and the variance of cell length as a function of magnesium sulfate concentration are shown in Figure 4A,B, respectively. Both the mean and the variance of the bacterial cell length are non-monotonic with a maximum at 1.25 M concentration of salt. These results suggest that the cell morphology is slightly affected at the salt concentrations below 0.83 M. Cell morphology exhibits a large heterogeneity at 1.25 M concentration of magnesium sulfate. Filamentous cells contribute to the increase of the average length of the cells, while both filamentous and short cells contribute to the variance of the cells at the concentration of 1.25 M. Therefore, both the mean and variance exhibit maximum at this concentration. Note that for concentrations >1.25 M, cells do not exhibit growth, and both dead and live cells exist in the population over the time scale of our experiments.

### 3.3. Plasmolysis of Cells

**Figure 5:**
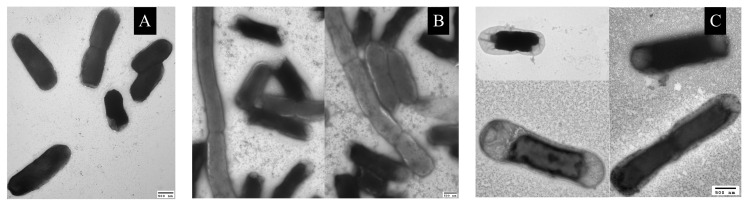
TEM images of *E. coli* at MgSO${}_{4}$ concentrations of (**A**) 2 mM, (**B**) 1.25 M, and (**C**) 2.07 M, respectively. Cells undergo plasmolysis at high salt concentrations and the extent of plasmolysis is greater near the poles.

Cells actively regulate their volumes in order to maintain the osmotic balance between the inside and the outside of a cell. If the osmolarity of the external environment is high, cells expel out the water from the cytoplasm to balance the osmotic pressure difference between the inside and outside of a cell. The loss of water from the cells causes the decrease of turgor pressure inside and under extreme osmotic stress, plasma membrane may detach from the cell-wall, leading to plasmolysis of cells. Earlier studies have suggested that *E. coli* may undergo plasmolysis under hyperosmotic conditions [39]. As we have discussed above, as the concentration of magnesium sulfate in the media increases, we also find cells smaller in length as compared to control cells, suggesting the loss of water from the cells. To investigate the nature of shortening of cells, we performed imaging of cells under a TEM. In Figure 5A–C are the representative TEM images of cells at 2 mM, 1.25 M, and 2.5 M magnesium sulfate, respectively. The scale bar is 500 nm. Plasmolysis of cells is clearly seen at high salt concentrations and the extent of plasmolysis is greater in the regions around the poles.

### 3.4. Expression of aqpZ, corA, cysP, and osmC

**Figure 6:**
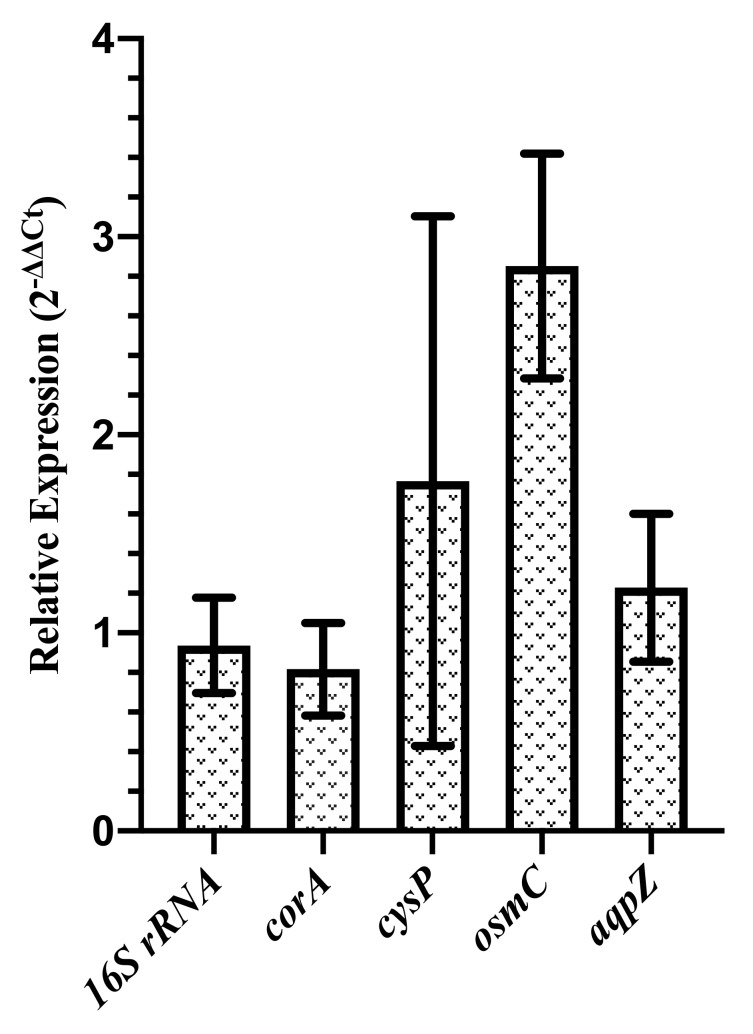
Relative expression, $2^{-\Delta \Delta \mathrm{Ct}}$, of the cells grown at 1.25 M magnesium sulfate as compared to cells grown in control media (2 mM MgSO${}_{4}$). Gene encoding the sulfate transporter, *cysP*, and an osmotically inducible gene, *osmC*, are upregulated at high concentration of magnesium sulfate. The expression of *cysP* exhibits large variability across biological replicates.

We next investigated the expression of genes involved in the osmotic transport of water (*aqpZ*), magnesium transport (*corA*), sulfate transport (*cysP*), and an osmotically inducible gene (*osmC*). We compare the expression of these genes between the cells grown at 2 mM and 1.25 M salt concentration. In Figure 6, we show the fold expression changes, $2^{-\Delta \Delta \mathrm{Ct}}$, of these genes between control (2 mM MgSO${}_{4}$) and cells grown at 1.25 M magnesium sulfate. The data is obtained by averaging over three biological and two technical replicates. The error bars were estimated from the standard error on ΔCt. Our results suggest that while the expressions of *aqpZ* and *corA* remain unchanged, the expressions of *cysP* and *osmC* are upregulated at 1.25 M salt concentration. Moreover, we observe a larger variability in expression of *cysP* across biological replicates. Interestingly, the expression of *aqpZ* does not change for the cells grown at high concentration of magnesium sulfate, as opposed to its downregulation in the exponential growth phase observed in the presence of hyperosmolar media containing NaCl [59]. However, later studies have found no role of *aqpZ* in the water permeability during the exponential growth phase but only during the stationary phase [60,61]. Since we measure the gene expression during the exponential growth phase, our result of no changes in *aqpZ* expression at high salt concentration is consistent with the results of Soupene et. al. [60]. The slow diffusion of water is still possible over long times, and therefore the plasmolysis observed at high salt concentration may result from slow diffusion of water into external media.

### 3.5. Reversibility of Population Doubling Time upon Removing the Applied Salinity Stress

**Figure 7:**
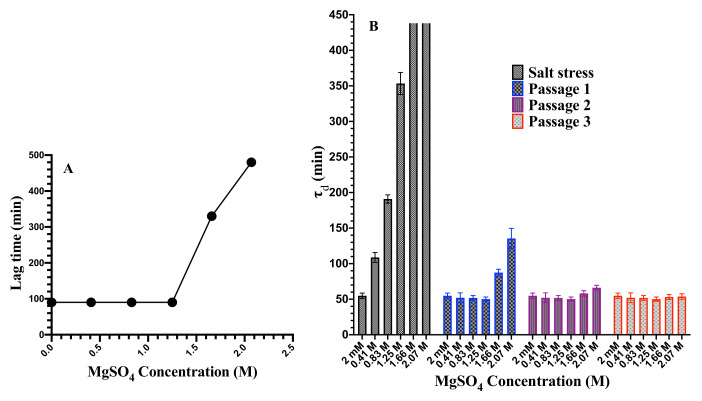
(**A**) Lag time as a function of magnesium sulfate concentration. The lag time increases with increasing concentration. (**B**) Population doubling time, $\tau_{d}$, during different passages after the removal of high salt stress. Open bars (1.66 M and 2.07 M) represent no growth. The population doubling time for the control cells grown at 2 mM MgSO${}_{4}$ is 55±5 min. The error on the $\tau_{d}$ is estimated from the instrumentation error in the measurement of optical density as described in the text.

We next asked questions regarding the reversibility of cell morphology and cell growth rate upon removal of high salt stress. In other words, how many generations does it take for the cells grown at high salt concentration before they start behaving like control cells upon the removal of salt stress? Cell morphology of bacteria grown under different environmental stresses, such as high hydrostatic pressure [31,62], temperature [63], and antibiotics [64,65] has been investigated. These studies have found filamentous cell morphology, similar to our observation of cell morphology at 1.25 M magnesium sulfate. This change in morphology is implicated in survival strategy [66]. Some of these investigations have focused on the reversibility dynamics of filamentous cells to normal cells [32]. A high concentration of magnesium sulfate leads to changes in morphology, not only due to lack of cell division but also due to partial plasmolysis of cells, as discussed above. Here, we focus on the time scale of recovery of cells once the salt stress is removed. The reversibility experiments were performed as described in the Materials and Methods section. For the first passage (right after the removal of salt stress), there was a significant lag phase before cells started to grow exponentially for all the samples grown at different concentrations of magnesium sulfate. We find that the lag time increases with the increasing concentration of magnesium sulfate (Figure 7A). The population doubling time, $\tau_{d}$, is also measured during each passage. Since the population doubling time changes with the number of passages (or generations), the $\tau_{d}$ obtained here is an average doubling time over five generations. Figure 7B shows the population doubling time, $\tau_{d}$, of cells at different salt concentrations as well as between 0–5 (passage 1), 5–10 (passage 2), 10–15 (passage 3) generations after removal of the salt stress. Here, the 0^th^-generation (passage 1) means the time when the cells first entered the exponential growth phase right after the removal of the salt stress. The error on $\tau_{d}$ is estimated from the instrumentation error, $\Delta_{\mathrm{OD}}=0.02$, on the measurement of optical density. We assumed a Gaussian statistics for the error $\Delta_{\mathrm{OD}}$ measured at each time point of a growth curve. We generated 1000 values of $\Delta_{\mathrm{OD}}$ for each time point of a growth curve from a Gaussian with a constant standard deviation $\Delta_{\mathrm{OD}}=0.02$ and fit exponentials through them. This gives us a set of 1000 values of population doubling time for each growth curve. The average of these values of population doubling times is reported as $\tau_{d}$ and the standard deviation of these population doubling times is taken as the error on $\tau_{d}$. Cell morphology was reversible and comparable to morphology of the control cells within five generations for all the salt concentrations studied here but the growth rate reversibility was dependent on salt concentration. Cells grown in the media containing ≤0.83 M MgSO${}_{4}$ are reversible in their growth rate in less than five generations after removing stress. Cells stressed under >0.83 M magnesium sulfate took a longer time before $\tau_{d}$ became comparable to that of the control cells. Cells grown at 1.66 M and 2.07 M concentration of magnesium sulfate, where a fractions of cells in a population undergo death or partial plasmolysis, reverted to the normal growth rate after ≈10 generations. These results suggest that the effect of high concentrations of magnesium sulfate can propagate over many generations even after the removal of the salt stress.

## 4. Discussion

In summary, we studied the effect of high concentrations of magnesium sulfate, presumably the most abundant salt on Europa and Mars, on cell growth, death, cell division, and gene expression of a halotolerant bacterium *E. coli*. The growth rate of cells decreases with salt concentration up to 1.25 M without any death. The result of the decrease of the growth rate with magnesium sulfate concentration is similar to the effect of other stressors on *E. coli*, such as high hydrostatic pressure [31,67], high and low temperatures [68], and extremes of pH [69]. For salt concentrations above 1.25 M, cells do not exhibit any growth and the cell death rate increases sharply. It is known that the viability of *E. coli* decreases upon increasing sodium chloride concentration beyond 0.51 M [37]. However, our data suggests that the cells are viable up to 1.25 M magnesium sulfate. Water activity is one of the major factors determining the growth and viability of cells. In Table 2, we list the values of water activity of aqueous solutions of magnesium sulfate [70], and compare it with the water activity of aqueous solutions of sodium chloride [71,72]. The water activity corresponding to the onset of cell death in magnesium sulfate is slightly smaller (0.974) as compared to the onset of cell death in sodium chloride (0.983). These values of water activity are significantly larger than the limiting value of water activity ≈0.6 [36] to support life. Our results suggest that *E. coli* can be viable in a much larger ionic strength of MgSO${}_{4}$, as compared to NaCl. It appears that in this case, the nature of the ions is the limiting factor and not the water activity.

We also investigated cell division and cell morphology. We find that for salt concentrations up to 0.83 M, the average cell length decreases. At 1.25 M of salt, cells exhibit three distinct morphologies—(i) filamentous cells, (ii) shorter than the control cells, and (iii) cells with a length comparable to control cells. The filamentous cells in the population arise due to the lack of cell division. TEM images of the cells revealed that cells undergo plasmolysis at high salt concentrations, with the plasmolysis being stronger in the regions around the poles. The heterogeneities in morphology and survival may arise due to underlying stochasticity in cellular processes, including gene expression [73], cell division [74], protein-protein interaction [75], mRNA synthesis and degradation [76], and stochastic nature of biochemical networks [77]. Cells grown at high salt concentrations were able to revert back to normal growth rate and morphology. The reversibility time of the growth rate increases with salt concentration. The dynamics of cell division of the filamentous cells during reversibility will be a subject of future investigation. Finally, we find that the expressions of *osmC* and *cysP* increase at salt concentration of 1.25 M. CysP, a thiosulfate binding protein, is a part of ABC transporter that regulates the transport of sulfate. Increased expression of *cysP* at high salt concentration suggests that cells control the uptake of sulfate by upregulating the expression of *cysP*. The expression of *osmC*, an osmotically inducible gene, also increases. The upregulation of *osmC* is consistent with what is found for the cellular response in other hyperosmolar environment [42]. *osmC* expression in *E. coli* is regulated by a number of genes. *osmC* is transcribed from two overlapping promoters, *osmC*${}_{p1}$ and *osmC${}_{p2}$* [78]. The transcription factor RpoS is needed for the transcription from promoter *osmC*${}_{p2}$, while it is transcribed from *osmC${}_{p1}$* in a RpoS-independent way [42,78]. Furthermore, the expression of *osmC* is regulated by a histone-like nucleoid structuring protein, H-NS, and leucine-responsive regulatory protein, Lrp [78,79]. Lrp represses the expression of *osmC* by binding to the promoter *osmC*${}_{p2}$, while H-NS represses both the promoters through an unknown mechanism. In the light of our results, it appears that the upregulation of *osmC* in the presence of a high salt concentration may arise from the increased production of RpoS and the transcription of *osmC* from *osmC${}_{p2}$* or in a RpoS-independent way from *osmC${}_{p1}$* during the exponential growth phase. Another possibility is the downregulation of H-NS and LrP or decreased binding affinities to bind to *osmC* promoters. We find that the expression level of *aqpZ* does not change at a high salt concentration, consistent with earlier studies of expression of *aqpZ* under hyperosmotic condition in the exponential growth phase [60,61].

Aqueous solutions of MgSO${}_{4}$ up to 1.25 M may exist at subzero temperatures in near-surface regions on Mars. Since *E. coli* can be viable up to 1.25 M of magnesium sulfate, our results are important for planetary protection. Besides Europa and Mars, our studies are also relevant for the habitability of other planetary bodies such as Ceres and Enceladus. The conditions on Europa do not only include high concentrations of magnesium sulfate but also high pressure and low temperature. We only investigated the behavior of the cells in one of the variables, salt concentration. Further studies, in which combinations of these environmental variables are considered, must be carried out in order to understand cellular responses of *E. coli* in those conditions.

## Figures and Tables

**Table 1:** List of the primers used.

Gene	Primer	Sequence (5${}^{\prime }$–3${}^{\prime }$)
16S *rRNA*	Forward	TCGTCAGCTCGTGTTGTGAA
	Reverse	AGGGCCATGATGACTTGACG
*corA*	Forward	AACATCGAGCAGAACCGCAT
	Reverse	AAAGATAATCGCGCCAGGGT
*cysP*	Forward	CGCCGTTTGAGCAACAATGG
	Reverse	TTTGTACGTCGGTCACCTGG
*osmC*	Forward	ATCGATTGATACCACCGCCG
	Reverse	GGGCATCCTGCTTTTGCTTT
*aqpZ*	Forward	AGCATTCACCAGGCGGTTAT
	Reverse	TCAGGGTTAAGGCCAGACCA

**Table 2:** Water activities of aqueous solutions of MgSO${}_{4}$ and NaCl at T = 25 °C. The data are taken from References [70,71,72].

Concentration (M)	Water Activity	
	MgSO${}_{4}$	NaCl
0.1	0.9970	0.9967
0.2	0.9950	0.9934
0.3	0.9940	0.9901
0.4	0.9920	0.9868
0.5	0.9900	0.9836
0.6	0.9880	0.9803
0.7	0.9870	0.9769
0.8	0.9850	0.9736
0.9	0.9830	0.9702
1.0	0.9810	0.9669
1.2	0.9760	0.9601
1.25	0.9740	0.9552
1.4	0.9710	0.9532
1.6	0.9660	0.9461
1.8	0.9600	0.9389
2.0	0.9530	0.9316
2.5	0.9320	0.9127
3.0	0.9050	0.8932
